# Posttraumatic Stress and Attentional Bias towards Cancer-Related Stimuli in Parents of Children Recently Diagnosed with Cancer

**DOI:** 10.1371/journal.pone.0152778

**Published:** 2016-04-01

**Authors:** Martin Cernvall, Emma Hovén, Lisa Ljungman, Gustaf Ljungman, Per Carlbring, Louise von Essen

**Affiliations:** 1 Clinical Psychology in Healthcare, Department of Public Health and Caring Sciences, Uppsala University, Uppsala, Sweden; 2 Pediatric Oncology, Department of Women’s and Children’s Health, Uppsala University, Uppsala, Sweden; 3 Department of Psychology, Stockholm University, Stockholm, Sweden; University of Stellenbosch, SOUTH AFRICA

## Abstract

**Objectives:**

To investigate whether posttraumatic stress symptoms (PTSS) are related to attentional bias towards cancer-related stimuli among parents of children recently diagnosed with cancer.

**Methods:**

Sixty-two parents completed questionnaires measuring PTSS, depression, and anxiety and the emotional Stroop task via the Internet. The emotional Stroop task included cancer-related words, cardiovascular disease-related words, and neutral words.

**Results:**

Participants were split in two groups based on the median of PTSS: High-PTSS and Low-PTSS. There was a significant interaction between word-type and group and a planned contrast test of this interaction indicated that the High-PTSS group had longer response latencies on cancer-related words compared to the other word-type and group combinations.

**Conclusions:**

Findings suggest that PTSS are related to attentional bias towards cancer-related stimuli among parents of children recently diagnosed with cancer. Implications of this finding for the understanding of PTSS in this population, future research, and clinical practice are discussed.

## Introduction

Being a parent of a child recently diagnosed with cancer is a stressful experience and research shows that these parents report a higher level of posttraumatic stress symptoms (PTSS) and are assessed with a higher frequency of a formal diagnosis of posttraumatic stress disorder (PTSD) compared to parents of children later during the disease trajectory [1–4], and compared to parents of healthy children [5, 6]. Others have shown that PTSS are positively associated with symptoms of depression [7] and anxiety [8]. Longitudinal studies indicate that parents typically report a level of PTSS in the moderate to severe range shortly after the child´s diagnosis, with a declining level over time [9, 10]. In addition, there is evidence of distinct subgroups with different development trajectories such as high-declining, moderate-stable, and low-stable distress levels during the child’s treatment [9]. Even though research suggests that symptoms decline with time since diagnosis there is also evidence of a subgroup reporting distress years after end of treatment [11].

Despite the growing literature on PTSS/PTSD in parents of children diagnosed with cancer, little is known about the cognitive processes related to these symptoms. Increased knowledge regarding the role of cognitive processes in the onset and maintenance of PTSS/PTSD, and other types of emotional distress among these parents, could inform research and clinical practice within the field of pediatric psychosocial oncology. It has been suggested that the prevalence of PTSS/PTSD in childhood cancer populations has been overestimated due to focusing effects [12]. Focusing effects refer to when people are cued to a specific event before they are asked to reflect and evaluate their internal state in relation to this event, which can impact their perception of their psychological state. Research that investigate whether PTSS/PTSD covaries with performance based measures that tap into cognitive processes thought to influence PTSS/PTSD, rather than only relying on self-reports, could elucidate the phenomena and construct validity of PTSS/PTSD in the current population.

One well-investigated cognitive process in relation to PTSS/PTSD is attentional bias, i.e., differential attentional allocation towards threatening compared to neutral stimuli [13]. One method to assess attentional bias is the emotional Stroop task [14] which exposes participants to stimuli and asks them to name the color of these. Response latencies are measured and attentional bias is assumed to occur if the participant takes longer time to identify the colors of certain stimuli (e.g., threat-related) compared to others (e.g., neutral). Studies that have employed the emotional Stroop task have shown that PTSS/PTSD is associated with attentional bias towards trauma-related stimuli among e.g., rape victims [15], survivors of traffic accidents [16, 17], and combat veterans [18, 19].

There are several, somewhat disparate, theories of the nature and mechanisms involved in attentional bias, see Cisler and Koster 2010 for a review [13]. However, one general view is that biased cognition plays a key role in emotional regulation such that people with a heightened proneness to emotional dysfunction respond to negative emotional states with biased patterns of cognition favoring the processing of negative information. This intensifies the negative emotional state, fueling a maladaptive emotion regulation cycle [20]. In PTSS/PTSD, attention is drawn to stimuli that remind of past trauma and consequently exacerbate the fear of future similar events, and this might be more pronounced in individuals with difficulties in emotional regulation.

To our knowledge there is only one study that has investigated attentional bias with the emotional Stroop task and PTSS in parents of children diagnosed with cancer [21]. The results did not indicate a relationship between PTSS and attentional bias towards trauma-related stimuli in parents of children diagnosed with cancer. However, the sample size was small and less than optimal words may have been used to detect a relationship. Only five words were used (i.e., *cancer*, *I*.*V*., *counts*, *brovie*, *chemo*), some which have wide usage outside the cancer context. Using generic cancer-related words other research has revealed that women with a family history of cancer exhibit attentional bias towards these words in comparison with women without such a history [22].

The Internet provides opportunities for data collection in research. Linnman and colleagues [23] developed an emotional Stroop task that can be administered via the Internet and showed that it produced comparable results as a conventional computerized version. This version of the Stroop task has been used in research on individuals with tinnitus [24], social anxiety disorder [25], eating disorders [26], and students participating in a sleep monitoring study [27].

In this study the Internet-based Stroop task developed by Linnman et al. [23] was adapted to assess attentional bias towards cancer-related stimuli among parents of children recently diagnosed with cancer. Based on previous findings it was hypothesized that level of PTSS would be associated with response latencies when identifying emotional material, such that parents reporting a higher level of PTSS would exhibit longer response latencies when identifying colors of cancer-related stimuli compared to neutral stimuli, and that this effect would not be present among parents reporting a lower level of PTSS. In order to control for the specificity of threat among cancer-related stimuli, words related to cardiovascular disease (CVD) were included. It was expected that neither parents reporting a high nor a low level of PTSS would have longer response latencies towards CVD-related words compared to neutral words.

## Materials and Methods

### Procedure and Participants

The data were collected during the screening phase of a randomized controlled trial (RCT) investigating the efficacy of Internet-based guided self-help for parents of children recently diagnosed with cancer [28]. During the screening phase participants completed self-report questionnaires and the Stroop task via the Internet. Swedish speaking parents of children on treatment for any type of cancer disease with access to a computer with an Internet-connection were potential participants. To be included in the RCT participants had to meet the modified symptom criteria on the PTSD-Checklist Civilian Version (PCL-C) [29], a self-report instrument corresponding to the Diagnostic and Statistical Manual of Mental Disorders– 4^th^ ed. (DSM-IV) model of PTSD [30], and not suffer from a psychiatric disorder in immediate need for treatment. The modified symptom criteria constitutes scoring ≥3 on at least 1/5 symptoms of re-experiencing, 1/7 symptoms of avoidance, and 1/5 symptoms of hyper-arousal, corresponding to partial PTSD [31]. However, all data collected from parents participating in the screening phase were analysed and reported in this study. Potential participants were approached at five of the six Swedish pediatric oncology centers and asked to participate by a nurse or physician 4–12 weeks after their child’s diagnosis. In the initial protocol potential participants were to be approached 1–2 weeks after diagnosis. However, during the first months of inclusion it was evident that parents were approached later after diagnosis due to administrative reasons, and the protocol was changed to the time frame reported. Individuals who consented were directed to a website where they could register to the study. Subsequent to registration, potential participants were e-mailed a unique code and link to a website where they could log in and complete questionnaires. Thereafter they were provided the option to complete the emotional Stroop task. The procedure was approved by the regional ethics review board in Uppsala (Dnr 2008/238), and all participants provided written informed consent.

One-hundred parents were screened for participation in the RCT (see Cernvall et al., 2015 [28] for further details), 62 of these completed the emotional Stroop task. Reason for not completing the task was limited exclusively to technical issues such as having a Macintosh computer rather than a PC. There were no differences between individuals completing vs. not completing the Stroop task in terms of PTSS, depression, anxiety, or demographic characteristics (*p*’s ranging from 0.11 to 0.92) except for age which was higher among completers (∆ = 3.2, 95%CI = 0.3–6.1, *p* < 0.05). Demographic and clinical characteristics for those completing the emotional Stroop task are presented in Table 1.

**Table 1:** Demographic and Clinical Characteristics for the Participants and their Children.

	Full sample	High-PTSS	Low-PTSS	
	(*n* = 62)	(*n* = 31)	(*n* = 31)	*p*
**Mothers *n* (%)**	36 (58)	21 (68)	15 (48)	0.12
**Age mean (*SD*)**	40.2 (7.2)	39.8 (8.1)	40.6 (6.5)	0.66
**University education *n* (%)**	31 (50)	15 (48)	16 (52)	0.79
**Employed *n* (%)**	51 (82)	25 (81)	26 (84)	0.74
**Living with child’s biological parent *n* (%)**	51 (82)	25 (81)	26 (84)	0.74
**Experience of previous traumatic event *n* (%)**	32 (52)	14 (45)	18 (58)	0.31
**Child age mean (*SD*)**	8.2 (5.4)	8.7 (5.2)	7.8 (5.6)	0.48
**Female child *n* (%)**	37 (60)	18 (58)	19 (61)	0.80
**Months since dx median (IQR)**	3 (2.8)	3 (2.5)	3 (3)	0.94
**Diagnosis *n* (%)**				0.49
Leukemia	28 (45)	12 (39)	16 (52)	
Sarcoma	11 (18)	6 (19)	5 (16)	
Lymphoma	6 (10)	5 (16)	1 (3)	
CNS-tumor	9 (15)	4 (13)	6 (16)	
Other malignancy	8 (13)	4 (13)	4 (13)	
**PCL-C mean (SD)**	42.1 (13.2)	52.5 (9.6)	31.7 (6.4)	<0.001
**BDI mean (SD)**	17.6 (9.5)	22.5 (7.2)	12.7 (9.1)	<0.001
**BAI mean (SD)**	11.7 (7.8)	16.3 (7.6)	7.1 (4.8)	<0.001

PCL-C = PTSD-Checklist Civilian Version, BDI-II = Beck Depression Inventory-II, BAI = Beck Anxiety Inventory, dx = diagnosis

### Measures

#### The emotional Stroop task

The emotional Stroop task deployed via the Internet was developed by Linnman and colleagues [23] and is based on Macromedia Flashplayer (version 6). The task was run via the Internet but response time measurement was done on the computer to assure that response latency was measured independently of the network connection or bandwidth. The program was written in Active Server Pages (version 3.0) and a MySQL-database (version 3.23.54) was used to store data. During the task words were displayed separately in lowercase letters against a black screen in one of four colors: red, blue, green, or yellow. Participants were instructed to ignore the meaning of the words and to focus on the colors in which the words were printed. Each word was displayed once in each color resulting in 192 trials for each participant. The order of words and colors were randomized but balanced for each participant. During the task, four gray boxes, upon which each of the color names were printed in black, were displayed on the computer screen. Participants were instructed to indicate their color-naming choice by clicking on the box representing the color name in which the stimulus was displayed as quickly as possible. Words were presented above the four color options and the location of each color option was randomized for each participant. To ensure that all trials begun at an equal distance from each color option, initiation of every new trial required the participant to click on a circle placed in the center of the four color options. If this was not done within 4 seconds of the last stimulus presentation, a reminder was displayed at the bottom left of the computer screen. To become familiar with the task, and to learn the location of each color option, participants were required to respond correctly 16 times during a practice session before the main trials were initiated. Words remained on the computer screen in both tasks until a response had been provided. The computer calculated the interval between stimulus onset and response to the nearest millisecond. The inter stimulus interval was 2 seconds. The test was completed in approximately 10 minutes.

Three types of words were used, 12 cancer-related words, 12 CVD-related words, and 24 neutral words. The cancer-related words were chosen based on a previous Stroop-study comparing attentional bias towards cancer-related stimuli in women with and without a family history of cancer [22]. Each of the neutral words was matched to one of the cancer- or CVD-related words in terms of number of letters and syllables. The words are displayed in the S1 Table.

### Self-report questionnaires

#### PTSD-Checklist Civilian Version

PTSS related to the child´s cancer disease were assessed with the PTSD-Checklist Civilian Version (PCL-C) [29]. The PCL-C consists of 17 items rated on a 5-point scale (1–5), corresponding to the items assessing the B, C, and D criteria in the DSM-IV. Ruggiero, Ben, Scotti, and Rabalais [32] report that the instrument has adequate internal consistency, test-retest reliability, and evidence for convergent and discriminant validity when compared to other well-established measures of PTSS, depression, and general anxiety. A value of 44 or above on the full scale suggests a diagnosis of PTSD [33]. Cronbach´s α in the current sample was 0.92.

#### Beck Depression Inventory-II

Depression was assessed with the Beck Depression Inventory-II (BDI-II) [34] consisting of 21 items rated on a 4-point scale (0–3). The BDI-II has shown good concurrent validity with its precursor BDI, the suggested cut-offs are: 0–13 indicating minimal, 14–19 mild, 20–28 moderate, and 29–63 severe depression. Cronbach´s α in the current sample was 0.90.

#### Beck Anxiety Inventory

Anxiety was assessed with the Beck Anxiety Inventory (BAI) [35] consisting of 21 items rated on a 4-point scale (0–3). The BAI has shown good test-retest reliability and convergent validity, suggested cut-offs are: 0–7 indicating minimal, 8–15 mild, 16–23 moderate, and 24–63 severe anxiety. Cronbach´s α in the current sample was 0.90.

### Statistical analyses

Independent t-tests, Wilcoxon singed rank test, Chi^2^-test, and Fisher´s exact test were used in preliminary analyses. A visual inspection of response latency scores revealed outliers and values exceeding 5000 milliseconds (ms) were coded as missing data (less than 1% of observations were larger than 5000 ms). In the main analyses, mixed effects modelling [36] was used taking into account the repeated nature of Stroop task data with each participant providing 192 response latency observations nested in the individual. Restricted maximum likelihood estimation was used incorporating available data and modelling missing data. Word-type was included as a random intercept. For the main analyses, continuous variables (age, PCL-C, BDI-II, BAI) were coded as binary variables by the means of median splits (lower = 0, higher = 1). Analyses were performed with IBM SPSS Statistics 20.

## Results

### Participants

The sample was split in two groups (i.e., High-PTSS and Low-PTSS) based on the median on the PCL-C (median 42.5). Demographic and clinical characteristics of the two groups are presented in Table 1. There were no differences between the groups in terms of demographic or child characteristics but participants in the High-PTSS group reported a higher level of PTSS, depression, and anxiety.

### Attentional bias

**Fig 1:**
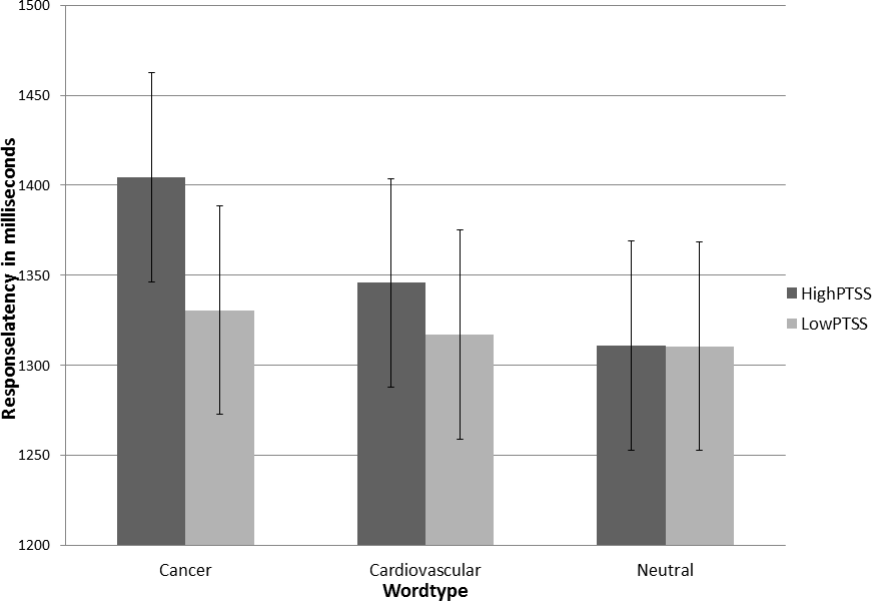
The effect of word-type and group on response latency in the emotional Stroop task (error bars represent standard errors [SE]).

None of the demographic or clinical variables were related to response latency. For the full sample there was a main effect of word-type, *F* (2, 227) = 9.01, *p* < 0.001, see Table 2. To test the main hypothesis, level of PTSS (High-PTSS vs. Low-PTSS) was included in the model which resulted in a significant interaction between word-type and group, *F* (2, 124.7) = 3.9, *p* < 0.05. A planned contrast test of the interaction showed that the High-PTSS group had longer response latencies on cancer-related words compared to the other word-type and group combinations, Estimate (141.8) = 59.4, SE = 24.2, *p* < 0.05, see Fig 1.

**Table 2:** Response Latencies in Milliseconds as a Function of Word-Type for the Full Sample.

	Word-Type		
	Cancer-related	CVD-related	Neutral
	*M* (*SE*)	*M* (*SE*)	*M* (*SE*)
**Full sample (*n* = 62)**	1368.2 (41.4)	1331.4 (41.4)	1310.7 (41.0)

CVD = cardiovascular disease.

There were high bivariate correlations between PTSS and depression (*r* = .69, *p* < 0.001) and between PTSS and anxiety (*r* = .78, *p* < 0.001). To examine the potential role of confounders in the relationship between PTSS, word-type, and response latency a series of exploratory analyses were conducted. Including depression or anxiety as a fixed effect only, either as a dichotomous (High vs. Low) or a continuous variable, did not affect the PTSS and word-type interaction. When testing the interaction between depression (High-Depression vs. Low-Depression) and word-type this interaction was non-significant, albeit on a trend-level, *F* (2, 128.2) = 2.7, *p* = 0.07. When including the PTSS and word-type interaction this resulted in a reduction of the depression and word-type interaction, *F* (2, 122.9) = 0.51, *p* = 0.58. The PTSS and word-type interaction was also non-significant, albeit with a higher *F* statistic, *F* (2, 119.7) = 1.7, *p* = 0.19. When testing the interaction between anxiety (High-Anxiety vs. Low-Anxiety) and word-type the interaction was non-significant, *F* (2, 124.7) = 0.33, *p* = 0.72. Altogether, these exploratory analyses suggest that PTSS had a stronger relationship with word-type in terms of response-latency than depression and anxiety, but that it was reduced when including the depression and word-type interaction, suggesting some overlap between the constructs. Finally, being a mother, having nine years or less education, being unemployed and being on sick-leave also interacted with word-type with longer response latencies for the cancer-related words. Therefore, these variables were included in similar exploratory analyses, however they did not reduce the PTSS and word-type interaction to non-significance in terms of response-latency.

## Discussion

The purpose of this study was to investigate whether parents of children recently diagnosed with cancer reporting a high level of PTSS show attentional bias towards cancer-related stimuli. The results supported this hypothesis as parents reporting a high level of PTSS had longer response latencies when identifying colors of cancer-related words compared to other word-types and parents reporting a low level of PTSS. This finding indicates that PTSS are associated with attentional bias towards cancer-related stimuli in parents of children recently diagnosed with cancer, and that this effect is specific to cancer-stimuli, not merely to disease-stimuli, as no such bias was evident towards CVD-related words.

The results relate to a large body of literature showing that anxious individuals show attentional bias towards threat-related stimuli compared to non-anxious individuals [37] and that attention bias is greater towards disorder-specific threat stimuli relative to disorder-non-specific threat stimuli [38]. Attention bias has been shown with various experimental methodologies such as the emotional Stroop task [14], the dot-probe paradigm [39], and the emotional spatial cuing paradigm [40] and in various populations with anxiety, e.g., PTSD, generalized anxiety disorder, panic disorder, and social phobia, see Bar-Haim et al. 2007 [37] for a review and meta-analysis. The results stand in contrast to the results presented by Hall and Baum [21] which showed no relationship between level of PTSS and attentional bias towards cancer-related stimuli among parents of children diagnosed with cancer. However, attentional bias towards cancer-related stimuli has been shown in other populations such as women with a family history of breast cancer [22] and attentional bias towards illness-related words has been shown in individuals with recent health problems [41]. Taken together the current findings and previous research show that cognitive processes such as those assessed with the emotional Stroop task provide information regarding the psychological processes at play in populations affected by disease or ill-health.

The results indicated an interaction between depression and word-type, albeit on a trend-level, and that the PTSS and word-type interaction was reduced when including the depression and word-type interaction. This suggests that there is overlap between PTSS and depression in the interaction with word-type and response-latency. This is not surprising given the high bivariate correlations between PTSS and depression in the current study and is also consistent with a large body of evidence showing that there is high comorbidity between PTSS/PTSD and depression indicating evidence for a shared vulnerability [42].

The results of the current study are preliminary and in need of independent replication, however some tentative implications can still be formulated. Firstly, the concept of PTSS/PTSD in parents of children with serious illness has been questioned on several grounds. On one hand it has been put forth that medical illness is not a sufficient event to elicit PTSD [43]. This is also reflected in the DSM-5 which suggests that adjustment disorder rather than PTSD should be used when describing the emotional impact and aftermath after medical illness [44]. On the other hand it has been argued that PTSS/PTSD is a valid concept among these parents but that prior studies may have overestimated the prevalence of PTSD in populations experiencing somatic illness [12]. This study indicates that level of PTSS differentiates individuals in terms of how they allocate attention to cancer-related stimuli. The results can elucidate the construct validity of PTSS in parents of children with serious illness such as cancer as it indicates that self-reported PTSS differentiate how participants perform on a performance based task that tap into cognitive processes thought to influence PTSS/PTSD. Performance based measures are less susceptible to factors such as focusing effects that have been suggested to inflate self-reported levels of PTSS/PTSD. Secondly, the results indicate that attentional bias should be incorporated in clinical conceptualizations of PTSS/PTSD that can guide interventions in parents of children with cancer. There is recent work suggesting that PTSS and related distress in these parents may be amplified and maintained by emotional regulating behaviors such as experiential avoidance and rumination in response to intrusive thoughts [45] and preliminary support for the efficacy of an intervention utilizing that conceptualization [28]. If the current results would be replicated in future studies it would indicate that this conceptualization should be updated with an explicit account of how experiential avoidance, rumination, and attentional bias affect PTSS/PTSD and related distress. Attentional bias has been conceptualized as an emotional regulating strategy and future research should investigate how different types of emotion regulation interplay in the onset and maintenance of PTSS/PTSD and distress in parents of children with serious illness.

Some limitations of the current study need consideration. Firstly, as the data collection was conducted via the Internet there was no way of controlling the setting in which participants completed the emotional Stroop task or the apparatus they used. On the other hand this may have enhanced the ecological validity of the study results. Secondly, data were collected at the screening/pre-assessment phase of a randomized controlled trial investigating the efficacy of guided self-help via the Internet. As such, the generalizability of the findings extends to parents of children recently diagnosed with cancer who experience a need and interest in participating in Internet interventions. Finally, it has been suggested that attentional bias as captured with the emotional Stroop task may constitute different attentional processes such as both automatic and conscious processes and differences in spatial attention allocation [13]. Future research in parents of children with serious illnesses should use methodologies that may tease these different processes apart.

## Conclusions

To conclude, the current study provides preliminary evidence that PTSS in parents of children recently diagnosed with cancer are related to attentional bias towards cancer-related stimuli. This tentatively elucidates the construct validity of PTSS in the population and findings can enhance the understanding of the distress that a proportion of these parents experience. If the current results are replicated, they can inform future research and clinical applications with this group.

## Supporting Information

S1 Table Stimulus Words with their English Translation. (DOCX)
