# Supplementary material for: Posttraumatic Stress and Attentional Bias towards Cancer-Related Stimuli in Parents of Children Recently Diagnosed with Cancer
Source: PLoS One. 2016 Apr 1;11(4):e0152778. doi: 10.1371/journal.pone.0152778 (PMC4817976; doi:10.1371/journal.pone.0152778)
Supplement: S1 Table — (DOCX) [file pone.0152778.s001.docx]

| **Cancer-related** | | **CVD-related** | | **Neutral** | |
| --- | --- | --- | --- | --- | --- |
| biopsi | (biopsy) |  |  | lamporna | ( the lamps) |
| cancer | (cancer) |  |  | soffbord | (coffe table) |
| cytostatika | (chemotherapy) |  |  | garderoberna | (the warderobes) |
| tumör | (tumor) |  |  | glaslådorna | (the glass drawers) |
| leukemi | (leukemia) |  |  | vaser | (vases) |
| malign | (malign) |  |  | bytta | (tub) |
| strålning | (radiation) |  |  | stolpen | (the pole) |
| knöl | (lump) |  |  | tall | (pine) |
| röntgen | (x-ray) |  |  | bänkrad | (row) |
| sjukdom | (disease) |  |  | mattor | (rugs) |
| operation | (operation) |  |  | bokhyllor | (bookshelves) |
| onkolog | (oncologist) |  |  | fåtöljen | (the armchair) |
|  |  | blockering | (blocking) | draperier | (draperies) |
|  |  | bypass | (bypass) | bomull | (cotton) |
|  |  | kardiolog | (cardiologist) | överkastet | (the bedspread) |
|  |  | kolesterol | (cholesterol) | kuddarna | (the pillows) |
|  |  | propp | (thrombus) | stropp | (strap) |
|  |  | infarkt | (infarction) | handduk | (towel) |
|  |  | hjärta | (heart) | filten | (the blanket) |
|  |  | blodtryck | (blood pressure) | gardin | (curtain) |
|  |  | pacemaker | (pacemaker) | glaskonsten | (the glass art) |
|  |  | stroke | (stroke) | lakan | (sheets) |
|  |  | aorta | (aorta) | oasen | (the oasis) |
|  |  | kranskärl | (coronaray artery) | bildduk | (white screen) |

CVD = cardiovascular disease.
